# Solitary fibrous tumor arising in the mesentery: a case report

**DOI:** 10.1186/1477-7819-9-140

**Published:** 2011-10-31

**Authors:** Sarah Bouhabel, Guy Leblanc, Jose Ferreira, Yves E Leclerc, Pierre Dubé, Lucas Sidéris

**Affiliations:** 1 Department of Surgery, Maisonneuve-Rosemont Hospital, University of Montreal, Montréal, Qc, 5415, boul. l'Assomption, H1T 2M4, Montréal, Canada; 2 Department of Pathology, Maisonneuve-Rosemont Hospital, University of Montreal, Montréal, Qc, 5415, boul. l'Assomption, H1T 2M4, Montréal, Canada

## Abstract

**Background:**

Solitary fibrous tumor (SFT) is a rare mesenchymal neoplasm usually found in the pleura, soft tissues and visceral organs. We describe one case arising in the mesentery, which is an exceptional localization.

**Case presentation:**

A 71-year-old man was referred to our establishment for a painless hypogastric mass. Further investigation revealed a vascular tumor, which was resected *en bloc*. Pathological findings confirmed solitary fibrous tumor of the mesentery.

**Conclusion:**

This is the second case of solitary fibrous tumor of the small intestine mesentery ever reported. It was managed by *en bloc *resection and close follow up considering the high risk of recurrence. Investigation should be made regarding the use of adjuvant systemic therapy to improve long-term survival for these patients.

## Background

Solitary fibrous tumor is a rare mesenchymal neoplasm first described in the pleura, in 1870, by Wagner [1]. Postulated cells of origin comprise fibroblasts, myofibroblasts endothelial cells, as well as pericytes [2]. It usually arises in the pleura, pericardium, soft tissues and visceral organs [2]. We report a case with an unusual localization in the mesentery. To our knowledge, this case would be the second reported case arising in the small intestine mesentery [3-7].

## Case presentation

**Figure 1:**
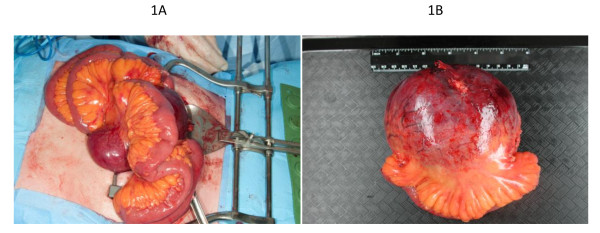
Solitary fibrous tumor of the mesentery.

A 71-year-old man was referred to the medical oncology department for the incidental finding of a palpable hypogastric mass. The patient did not complain of abdominal pain or any other gastrointestinal symptoms. He was in good general condition, with no relevant past medical history, except for a right total hip replacement that the patient underwent 10 years previously. A percutaneous biopsy was initially done to rule out a possible lymphoma. The pathology results were however consistent with a hemangiopericytoma or a solitary fibrous tumor. The patient was then referred to our surgical oncology department for resection. Abdominal CT scan with contrast revealed a fibrous tumor at the root of the mesentery, surrounding the superior mesenteric vein. There was neither thrombosis, nor hepatic or lymphatic involvement and the tumor seemed resectable. On March 12th 2010, the patient underwent surgery with complete resection of the mesenteric tumor as well as 22 cm of small intestine (Figure 1). The patient had an uneventful recovery and was discharged 6 days after surgery.

**Figure 2:**
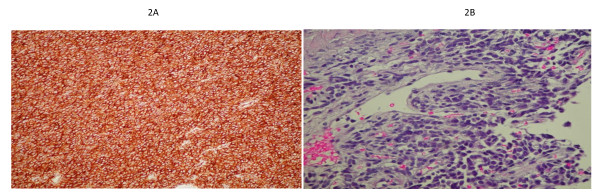
**2A. High diffuse positivity to anti-CD34**. 2B. Vessels revealing a *herringbone *pattern.

Gross pathologic examination revealed a 15,5 × 14 × 9 cm multinodular lesion. It was solid, pink-to-gray shaded and well vascularised without any macroscopic necrosis. It had no attachment to the intestinal wall, and presented an infiltrative pattern at the root of the mesentery. The small intestine portion was normal and the margins were clear. The specimen was submitted for histopathologic study, which was in favor of a malignant solitary fibrous tumor. Indeed, the tumor was more than 15 cm, and presented 6 mitoses per 10 high-power fields (HPF). Immunohistochemical studies revealed cells strongly positive to CD34 antibody (common in this type of neoplasm [5,8]) (Figure 2A), with focal CD99 membranous expression.

At twelve months of follow-up, there was no recurrence of the tumor.

## Discussion

Solitary fibrous tumor arising in the mesentery is an extremely unusual location for this type of tumor.

These tumors arise mostly in serosal surfaces of the pleura and pericardium [9]. Many extrapleural localizations have been previously reported including: retroperitoneum, deep soft tissues of proximal extremities (thigh, axilla), abdominal cavity, kidneys, trunk, and head and neck (including the orbit and meninges) [3] and only one previous case in the same localization [9]; mesentery of the small intestine.

Solitary fibrous tumors arise in patients with a median age of 40 to 60, and show no sex or race predominance. Our patient presented with a painless abdominal mass, which seems to be the case for most intra-abdominal SFT tumors [10].

From a strictly pathologic standpoint, great confusion exists in the literature regarding the origin, evolution and classification of solitary fibrous tumors. Indeed, solitary fibrous tumors and hemangiopericytomas (HPC) share many pathological findings. Overlapping nonspecific histologic features such as branching staghorn vessels, immunohistochemical CD34/CD99 reactivity, as well as ultrastructural pericytic, fibroblastic and/or myofibroblastic differentiation, renders difficult the differentiation of these tumors. The tendency is now one of considering HPC and SFT as being part of the same entity, with a broad morphological spectrum of differentiation.

Many tumors may contain areas mimicking SFT/HPC, therefore the differential diagnosis must be thoroughly reviewed and investigated. It comprises GIST, synovial sarcoma, leiomyosarcoma, undifferentiated liposarcoma, myofibroblastoma, metastasis from a spindle-cell carcinoma, malignant peripheral nerve sheath tumor (MPNST), as well as low grade fibromyxoid sarcoma [2,11].

Given that the specimen revealed cells strongly positive to CD34 antibody and focal CD99 membranous expression, the diagnosis was in favour of a solitary fibrous tumor [2,11]. Moreover, immunohistochemical study with CD117 marker was performed and did not show any reactivity, excluding the possibility of GIST. Bcl-2 antigen study (positive in 30% of SFTs [2]) was not done, since it was not required for the differential diagnosis. Extensive sampling did not reveal any lipoblastic differentiation, thus excluding dedifferentiated liposarcoma. Immunhistochemistry did not show any S-100 or SMA positivity eliminating the possibility of MPNST and leiomyosarcoma. Monophasic synovial sarcoma is at times difficult to distinguish from SFT. Diffuse expression of CD34 and absence of EMA favours SFT. Low grade myofibroblastic sarcomas usually show focal myxoid stroma and are normally CD34 negative.

The histopathologic study is important to establish prognosis of the tumor, since there is no grading scale *per se *for SFT [2]. Malignant nature is generally conveyed by a large tumor size (> 50 mm) [5,10,12] high cellularity, nuclear pleomorphism [11], mitotic activity (> 4 per 10 HPF), anaplasia, necrosis and hemorrhage [9,10]. In our case, the presence of nuclear polymorphism with hypercellularity, increased mitotic activity, tumor size, as well as a focally infiltrative interface are consistant with a malignant nature for this tumor.

Treatment of intra-abdominal SFT/HPC usually implies complete *en bloc *surgical resection with negative margins [9,10,13,14]. Radiotherapy is an adjunctive therapy that has been used in several patients. In our case, since the tumor was located centrally in the abdomen, surgical treatment without radiotherapy was favoured, in order to avoid the high risk of toxicity to the small bowel. The literature also reports the use of doxorubicin to achieve a partial response in patients with non resectable or advanced SFT's [10]. Use of interferon-alpha has also been reported, with resulting stabilization of the tumor [9]. However, no such reports exist for adjuvant systemic treatment.

Due to the rarity of solitary fibrous tumors in this particular location as well as the confusion regarding the pathological diagnosis, guidelines for treatment remain currently unclear. The outcome does not seem to be strongly related to the morphology of the tumor: even though tumors showing malignant features may behave aggressively, some tumors considered benign may recur and metastasize [2]. The prognosis of SFT is not well known, but the rate of recurrence and metastases is quite high (up to 50%) [10], occuring even years later (*ad *24 years, as reported by Rew and Allen in 1986 [15]). These tumors mainly spread haematogenously, and the lungs, liver and bone are the most common sites for metastases.

Solitary fibrous tumors are rare. Treatment consists in complete surgical resection. Considering that recurrence and metastases are common following surgery, long term follow up is necessary [13,5]. There is currently an unmet medical need regarding adjuvant treatment to try to improve long-term survival.

## Consent

Written informed consent was obtained from the patient for publication of this case report and the accompanying images.

## Competing interests

The authors declare that they have no competing interests.

## Authors' contributions

LS, GL and SB contributed to the Design of this case report; SB and LS contributed to the Redaction of the manuscript and to the Data acquisition; GL, JF, YL, PD and LS made a Critical review of this paper. All authors read and approved the final version of the manuscript.
